# Self-efficacy–kinesiophobia correlation in postoperative patients with primary liver cancer: the chain-mediating effect of postoperative fatigue and negative emotions

**DOI:** 10.3389/fpsyg.2025.1597999

**Published:** 2025-08-26

**Authors:** Zhijun Liu, Xingjuan Luo, Hui Yang, Mengqi Ma, Hong He, Hong Xiao

**Affiliations:** ^1^Department of Hepatobiliary and Pancreatic Surgery, Renmin Hospital, Hubei University of Medicine, Shiyan, Hubei, China; ^2^Medical Faculty, Wuhan University of Science and Technology, Wuhan, Hubei, China; ^3^School of Basic Medicine, Hubei University of Arts and Science, Xiangyang, Hubei, China; ^4^Nursing Department, Xiangyang Central Hospital, Affiliated Hospital of Hubei University of Arts and Science, Xiangyang, Hubei, China

**Keywords:** primary liver cancer, kinesiophobia, self-efficacy, postoperative fatigue, negative emotions, chain-mediating effect

## Abstract

**Introduction:**

Early postoperative mobilization poses a momentous challenge for patients with primary liver cancer (PLC) after undergoing laparoscopic surgery, and the onset of kinesiophobia can adversely affect their physical activity, mental health, and overall quality of life. This study aims to investigate the impact of self-efficacy on kinesiophobia in postoperative patients with PLC, along with the chain-mediating effect of postoperative fatigue (POF) and negative emotions on self-efficacy and kinesiophobia.

**Methods:**

This cross-sectional study used demographic questionnaires, the Tampa Scale for Kinesiophobia-11 (TSK-11), and the General Self-Efficacy Scale (GSES) to survey 328 patients who underwent laparoscopic surgery for PLC between January and June 2024.

**Results:**

There was a moderate negative correlation between self-efficacy and kinesiophobia in postoperative patients with PLC (*r* = −0.544, *p* < 0.01). POF mediated the self-efficacy–kinesiophobia correlation (= −0.159, 95% confidence interval (CI): [−0.248, −0.052]), accounting for 27.32% of the total effect. Moreover, negative emotions mediated the self-efficacy–kinesiophobia correlation (= −0.108, 95% CI: [−0.281, −0.026]), accounting for 18.56% of the total effect. Furthermore, the chain-mediating effect of POF and negative emotions on self-efficacy and kinesiophobia was significant (= −0.069, 95% CI: [−0.190, −0.019]), with the total indirect effect accounting for 57.56% of the total effect.

**Conclusion:**

The prevalence of kinesiophobia is comparatively higher among patients after PLC surgery. Self-efficacy not only directly affects kinesiophobia but also indirectly influences it through POF and negative emotions. This study offers a scientific basis for creating effective interventions to alleviate the adverse physiological and psychological effects of kinesiophobia in postoperative patients with PLC.

## 1 Introduction

Primary liver cancer (PLC) is one of the leading malignant tumors worldwide. China accounts for 45.3% of global PLC cases, ranking fourth in terms of new cancer cases and second in terms of mortality rate (Rumgay et al., 2022; Zheng et al., 2024). PLC is manifested by its high frequency, rapid progression, severe symptoms, and poor prognosis. Surgery remains the primary approach for treating PLC (Liu et al., 2024). Postoperatively, owing to tumor-related depletion and insufficient nutritional intake, patients often experience muscle weakness, physical debilitation, and fatigue, resulting in decreased mobility. Pertinent guidelines recommend that early postoperative mobilization is advantageous for decreasing complications and promoting recovery (Joliat et al., 2023). In addition, physical activity has been demonstrated to positively influence health outcomes, quality of life, and survival rates in patients with cancer (Elshahat et al., 2021). Likewise, Jeong et al. (2020) confirmed that promoting patient activity during hospitalization correlated with better outcome metrics and lower risk of hospital-related functional decline. Furthermore, Dennett et al. (2020) illustrated that physical activity aids in alleviating fatigue in patients with cancer.

Nevertheless, postoperative patients with PLC could experience varying degrees of visceral or incisional pain, excessive concern about early mobilization causing wound bleeding, drain dislodgement, or wound dehiscence, thereby diminishing their motivation for early mobilization and increasing fear of movement (a.k.a. kinesiophobia). Kinesiophobia is an irrational and excessive fear of physical activity attributed to persistent pain caused by movement (Kori, 1990). Inadequate postoperative activity in patients with PLC can cause complications like deferred gastrointestinal recovery, deep vein thrombosis, pulmonary infections, and intestinal adhesions. Reportedly, kinesiophobia correlates with slower recovery, extended hospitalization, and declined quality of life in postoperative patients (Wang and Du, 2024; Zeng Z. et al., 2024).

Self-efficacy is a key component of self-management, defined as an individual’s belief or confidence in their ability to accomplish specific behavioral goals in a particular domain. The level of self-efficacy signifies an individual’s confidence and motivation to overcome challenges in their current condition (Ruiz et al., 2024). Self-efficacy plays a vital role in disease recovery, with patients having higher self-efficacy often exhibiting better rehabilitation outcomes and enhanced quality of life (Bandura, 1977). Moreover, self-efficacy positively correlates with physical activity and behavioral changes in patients with cancer (Hoedjes et al., 2022). For postoperative patients with PLC, overcoming the fear of early mobilization and enthusiastically engaging in rehabilitation exercises are noteworthy challenges. Patients with low self-efficacy might struggle to complete postoperative rehabilitation activities successfully.

Previous research has established a self-efficacy–kinesiophobia correlation, suggesting that patients with higher self-efficacy display lower levels of kinesiophobia (Chen et al., 2025). In addition, some studies have demonstrated the indirect effects of self-efficacy on kinesiophobia, including the mediating role of positive coping strategies (Shi et al., 2023). However, most studies on kinesiophobia in patients with cancer focus on breast and lung cancer populations, with limited research on kinesiophobia in PLC. Thus, this study explores potential mediating variables between self-efficacy and kinesiophobia in patients with PLC.

Postoperative fatigue (POF) is a common physiological and psychological state experienced after surgery, reflecting a clinical manifestation of stress or trauma due to surgical procedures. POF is characterized by persistent physical and mental exhaustion and is affected by factors like surgical trauma, anesthesia, postoperative pain, and sleep disturbances. POF not only influences physical recovery but also accounts for psychological discomfort, manifesting as weakness, poor sleep quality, depression, and difficulty concentrating (Zheng et al., 2021); these symptoms could lead patients to avoid or completely disengage from physical activity, adversely affecting their functional recovery, mental health, and quality of life. Huang et al. (2023) suggested that POF can directly affect the incidence of kinesiophobia and, perhaps, indirectly affect kinesiophobia as a mediating variable. Postoperative patients with PLC often experience psychological reactions, such as anxiety and depression, which not only affect emotional states but also hinder recovery and quality of life. Previously, a significant positive correlation has been established among anxiety, depression, and kinesiophobia, with severer anxiety and depression correlating with a higher likelihood of kinesiophobia (Yakut Ozdemir et al., 2023).

The present research on the correlations among self-efficacy, kinesiophobia, POF, and anxiety/depression remains scant. Hence, this study aims to examine the correlations among self-efficacy, POF, anxiety/depression, and kinesiophobia in postoperative patients with PLC. The findings will offer a theoretical basis for clinical interventions aimed at improving mental health and quality of life in this population. Accordingly, this study proposed the following hypotheses: (i) POF mediates the relationship between self-efficacy and kinesiophobia; (ii) Negative emotions mediate the relationship between POF and kinesiophobia; and (iii) The impact of self-efficacy on kinesiophobia is mediated through a chain mechanism involving POF and negative emotions.

## 2 Materials and methods

### 2.1 Study sample

Using convenience sampling, we selected postoperative patients with PLC from six tertiary hospitals in Wuhan, Xiangyang, Jingzhou, and Shiyan, Hubei Province, from January to June 2024. The inclusion criteria were as follows: (i) aged ≥18 years, with no mental or cognitive impairments; (ii) diagnosed with PLC via imaging and treated with laparoscopic surgery; and (iii) aware of their condition and willing to participate in the study. Meanwhile, the exclusion criteria were as follows: (i) severe organ dysfunction; (ii) diseases limiting lower limb mobility; and (iii) a history of surgery or trauma in the lower limbs or conditions limiting lower limb activity.

The standardized recruitment procedure began with the screening of eligible patients in the electronic medical record system by attending physicians, followed by researcher-administered online questionnaires during the hospitalization period. After comprehensive disclosure of the study objectives, detailed procedures, and protection rights of the participants, written informed consent was obtained from all prospective participants, with formal enrollment only proceeding after the confirmed acquisition of consent.

Based on the sample size estimation principle, the sample size should be usually 10–20 times the number of independent variables. As this study included 18 independent variables, it required a sample size of 180–360. Considering a 20% invalid response rate, the calculated sample size was 216–432. Overall, 338 patients were contacted, but 10 refused to participate, mainly due to lack of interest or physical discomfort after surgery. Finally, we enrolled 328 patients. Notably, the Ethics Committee of Wuhan University of Science and Technology approved this study (Approval No.: 2024060), and we obtained informed consent from all participants.

### 2.2 Research tools

#### 2.2.1 Demographic questionnaire

Developed by the researchers based on the literature review and expert consultation, the questionnaire contained gender, age, education level, marital status, occupation, residence, monthly household income per capita, medical payment methods, whether the assistance of medical staff was needed during the first mobilization, and the presence of comorbidities.

#### 2.2.2 Tampa scale for kinesiophobia

The Tampa Scale for Kinesiophobia (TSK-11) was based on the fear-avoidance model, and later translated into Chinese by Cai et al. (2019). The scale contains 11 items categorized into three dimensions, namely, activity cognition, activity behavior, and activity attitude. It uses a 4-point Likert scale (1 = strongly disagree, 2 = disagree, 3 = agree, and 4 = strongly agree) and the total score ranges between 11 and 44, with a score >26 representing kinesiophobia; higher scores denote greater kinesiophobia. The scale has Cronbach α coefficients of 0.797 to 0.971 in breast cancer patients and a test–retest reliability of 0.499 to 0.938, illustrating good reliability and validity (Fernández-Gualda et al., 2024). Besides, its brevity, simplicity, and ease of understanding render it extensively applicable in clinical settings.

#### 2.2.3 General self-efficacy scale

The General Self-Efficacy Scale (GSES) is the most widely used measure of general self-efficacy worldwide. It was revised and translated into Chinese by Wang et al. (2001), which consists of 10 items rated on a 4-point Likert scale, with total scores ranging from 10 to 40; higher scores indicate greater self-efficacy. The scale has a Cronbach α coefficient of 0.95 in patients with colorectal cancer, indicating excellent internal consistency (Hong Hanh et al., 2024).

#### 2.2.4 Identity-consequence fatigue scale-10

The Identity-Consequence Fatigue Scale (ICFS) was developed by Paddison et al. (2006) to assess POF and initially comprised 31 items. After extracting 10 items from the ICFS, Nogueira et al. (2017) developed the ICFS-10, which was later translated into Chinese by Xu et al. (2021). The Chinese version includes two factors, namely, vitality and physical strength. The scale has a Cronbach coefficient of 0.928 in gastrointestinal tumor, thereby illustrating good reliability and validity.

#### 2.2.5 Hospital anxiety and depression scale

The Hospital Anxiety and Depression Scale (HADS) has two 7-item subscales assessing symptoms of anxiety (HADS-Anxiety) and depression (HADS-Depression), scored on 4-point Likert scales (0–3). The total score ranges between 0 and 21, with separate subscales for anxiety and depression. A score >7 denotes the presence of anxiety or depression, with higher scores indicating greater severity. Both the HADS-Depression and HADS-Anxiety subscales demonstrated strong internal consistency (Cronbach α coefficient >0.8) in patients with decompensated cirrhosis (Zeng C. et al., 2024).

### 2.3 Data collection and quality control

Led by a graduate supervisor as the quality control manager, the research team included 1 nursing graduate student, 1 hepatobiliary-pancreatic surgeon, and 3 nursing staff with senior titles or above. To ensure sample homogeneity across study sites, participants were recruited exclusively from tertiary Grade A hospitals with comparable infrastructures and service standards. All research personnel underwent standardized training to ensure adherence to the protocol, and preliminary pilot testing confirmed consistent implementation across all sites. This multi-faceted approach effectively minimized inter-hospital variability in data collection. Data collection was done through questionnaires from eligible patients within 1 week postoperatively. The questionnaires were circulated through social media platforms (e.g., WeChat and QQ) using Wenjuanxing. Data were collected through the Wenjuanxing platform. Before access to the questionnaire, all participants were required to review the electronic “Patient Informed Consent Form.” The system presented two explicit options: “Agree to participate” or “Decline to participate.” Only participants selecting “Agree to participate” option were automatically redirected to the formal questionnaire page, ensuring documented consent before data collection. For patients having difficulty understanding the items, the questionnaire administrator explained the items; the patients repeated the questions and answers, and the administrator recorded the responses to guarantee objectivity and validity. A total of 328 questionnaires were circulated and all returned. After excluding 10 invalid responses, 318 valid questionnaires were finalized, yielding an effective response rate of 96.95%.

### 2.4 Statistical analyses

All data were sorted and exported to Excel for analysis. Descriptive statistics and Pearson correlation analysis were performed using SPSS 26.0, a structural equation model (SEM) was constructed using AMOS 24.0, and mediation effects were tested using Bootstrap, with a significance level of = 0.05. The specific methods included in this study were as follows:

Descriptive statistics to analyze demographic data and scale scores. While normally distributed continuous variables are presented using mean standard deviation, categorical variables are described using frequencies and percentages.

One-way analysis of variance (ANOVA) to assess the impact of different demographic characteristics on postoperative kinesiophobia in patients with PLC.

Pearson correlation analysis to examine the correlations among kinesiophobia and self-efficacy, POF, anxiety, and depression.

SEM using AMOS, with model assessment and modification done through maximum likelihood estimation. In addition, Bootstrap was used to test the significance of mediation effects, with 95% confidence intervals (CI) calculated. If the 95% CIs did not include zero, the mediation effect was considered significant.

## 3 Results

### 3.1 Common method bias tests

Harman’s single-factor test revealed 11 factors with eigenvalues >1. The cumulative variance described by the first factor was 37.98%, which was below the critical threshold of 40.00%, thereby indicating that no significant common method bias existed in the questionnaire results of this study.

### 3.2 Descriptive statistics

Table 1 presents the descriptive statistical information of the study group, together with the TSK-11 scores. Significant differences in the TSK-11 scores were observed among patients with different educational levels, monthly household incomes, and medical payment methods, as well as whether they needed assistance from medical staff during their first mobilization (*p* < 0.05). The findings revealed that patients with educational levels at or below high school exhibited significantly higher kinesiophobia scores compared to their university-educated counterparts. Households with per capita monthly incomes exceeding 3,000 RMB showed elevated kinesiophobia levels compared to those with lower incomes. In terms healthcare financing, patients who were self-funded manifested more severe kinesiophobia than patients utilizing other payment modalities. Notably, patients who did not receive professional assistance during initial postoperative ambulation displayed substantially higher kinesiophobia scores than those who obtained clinician support.

**Table 1:** TSK-11 score in postoperative patients with primary liver cancer (*n* = 318).

Variable	Category	*n* (%)	TSK-11 (score, ^−^x ± s)	*t*/*F*	*P*
Sex	Female	152 (47.79)	28.79 ± 12.02	0.978	0.326
	Male	166 (52.21)	30.11 ± 11.75		
Age/years	18–40	20 (6.30)	31.90 ± 12.55	0.915	0.401
	41–65	122 (38.36)	30.12 ± 11.63		
	>66	176 (55.34)	28.76 ± 11.98		
Educational level	Illiterate	95 (29.87)	30.76 ± 11.34	5.305	0.000***
	Primary school	15 (4.72)	29.60 ± 11.83		
	Junior high school	81 (25.47)	32.84 ± 10.58		
	High school/trade school	113 (35.53)	27.10 ± 12.47		
	University and above	14 (4.40)	20.50 ± 10.65		
Marital status	Married	291 (91.51)	29.12 ± 11.95	1.619	0.200
	Unmarried	16 (5.03)	33.50 ± 10.49		
	Divorced/widowed	11 (3.46)	33.27 ± 10.95		
Occupation	Enterprises/civil servants	4 (1.18)	27.75 ± 12.87	1.731	0.143
	Workers/farmers	224 (70.68)	29.58 ± 11.91		
	Unemployed	18 (6.50)	33.83 ± 11.66		
	Freelance	13 (3.14)	22.62 ± 10.87		
	Retiree	59 (18.51)	29.41 ± 11.66		
Place of residence	Rural	168 (52.83)	29.33 ± 11.96	0.059	0.807
	Urban	150 (47.17)	29.65 ± 11.83		
Monthly household income per capita (RMB)	<1,000	36 (11.32)	26.61 ± 11.40	2.762	0.042*
	1,000–3,000	8 (2.52)	21.88 ± 10.93		
	3,001–5,000	142 (44.65)	29.09 ± 12.10		
	>5,000	132 (41.50)	31.15 ± 11.57		
Payment of medical costs	Commercial insurance	16 (5.03)	22.69 ± 12.56	3.632	0.013*
	Resident medical insurance	180 (56.60)	30.95 ± 11.42		
	Employee medical insurance	113 (35.53)	27.87 ± 12.03		
	Self-funded	9 (2.83)	32.44 ± 12.75		
First-time out-of-bed medical help	Yes	262 (82.39)	30.09 ± 11.85	1.979	0.049*
	No	56 (17.61)	26.64 ± 11.71		
Comorbidity with other diseases	Yes	18 (5.66)	29.22 ± 12.92		
	No	179 (56.28)	29.64 ± 11.78	0.035	0.965
	Unknown	121 (38.05)	29.29 ± 11.97		

TSK-11, Tampa Scale for Kinesiophobia. *p < 0.05.

### 3.3 Correlations among main variables

Table 2 demonstrates a negative correlation between kinesiophobia and self-efficacy (*r* = −0.541, *p* < 0.01), whereas a positive correlation with POF (*r* = 0.606, *p* < 0.01), anxiety (*r* = 0.328, *p* < 0.01), and depression (*r* = 0.401, *p* < 0.01).

**Table 2:** Means, standard deviations, and Spearman correlations among TSK-11, GSES, POF, and HADS.

Variable	Mean value	Standard deviation	Kinesiophobia	Self-efficacy	Postoperative fatigue	Anxiety	Depression
Kinesiophobia	29.481	11.879	1	–	–	–	–
Self-efficacy	24.758	10.211	−0.544**	1	–	–	–
Postoperative fatigue	37.635	11.724	0.606**	−0.446**	1	–	–
Anxiety	18.560	8.194	0.328**	−0.381**	0.380**	1	–
Depression	18.808	7.703	0.401**	−0.390**	0.377**	0.389**	1

GSES, General Self-Efficacy Scale; HADS, Hospital Anxiety and Depression Scale; POF, postoperative fatigue; TSK-11, Tampa Scale for Kinesiophobia. **p < 0.01.

### 3.4 Multiple linear regression analysis

The multiple linear regression analysis was performed using kinesiophobia as the dependent variable and education level, monthly household income, medical payment method, assistance during the first mobilization, self-efficacy, POF, and anxiety/depression as independent variables. The results demonstrated that education level, monthly household income, assistance during the first mobilization, self-efficacy, POF, and anxiety/depression were significant predictors in the regression model, explaining 49.2% of the total variance (Table 3).

**Table 3:** Multiple linear regression analysis of kinesiophobia (*n* = 318).

	Nonstandardized coefficients		Standardized coefficient	*t*	*P*	Covariance diagnosis	
	Exp (B)	SE	β			VIF	Tolerance
Constant	16.220	4.680	–	3.466	0.001	–	–
Educational level	−1.191	0.369	−0.132	−3.225	0.001	1.048	0.954
Monthly household income per capita	−1.147	0.574	−0.090	−1.996	0.047	1.268	0.789
Payment of medical costs	0.831	0.771	0.044	1.079	0.282	1.023	0.978
First-time out-of-bed medical help	2.845	1.391	0.091	2.045	0.042	1.246	0.802
Self-efficacy	−0.330	0.056	−0.284	−5.922	0.00	1.436	0.696
Postoperative fatigue	0.460	0.051	0.454	9.049	0.00	1.571	0.637
Anxiety	1.033	0.463	0.102	2.233	0.026	1.298	0.770
Depression	1.033	0.512	0.105	2.209	0.028	1.407	0.711

*R*^2^ = 0.505, adjusted *R*^2^ = 0.492.

### 3.5 Chain-mediating effect of self-efficacy on kinesiophobia

**Figure 1:**
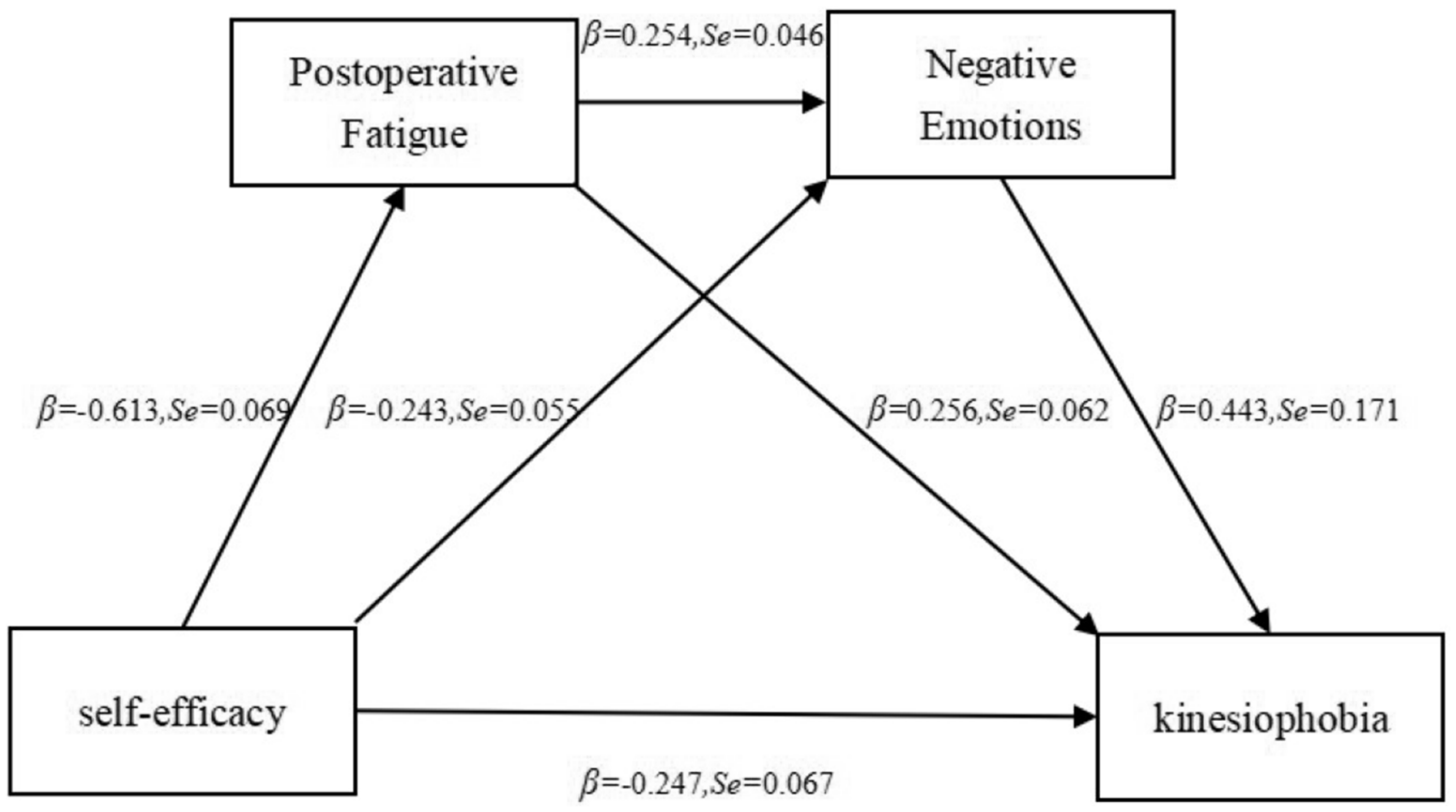
Mediation effect model of postoperative fatigue and negative emotions in the self-efficacy–kinesiophobia correlation.

An initial SEM was constructed with self-efficacy as the independent variable, kinesiophobia as the dependent variable, and POF and negative emotions as mediating variables. The structural equation model demonstrated an excellent fit: χ^2^/DF = 1.796, NFI = 0.96, IFI = 0.982, GFI = 0.932, CFI = 0.982, RFI = 0.952, AGFI = 0.907, and RMSEA = 0.051. Figure 1 presents the final SEM. Using Bootstrap, we tested the significance of the mediating effects of POF and negative emotions on self-efficacy and kinesiophobia. With 5,000 repeated samples, 95% CIs were calculated; if the 95% CIs did not include zero, the mediating effects were considered significant.

Table 4 illustrates that POF mediates the self-efficacy–kinesiophobia correlation (= −0.159, 95% CI: [−0.248, −0.052]); negative emotions mediate the self-efficacy–kinesiophobia correlation (= − 0.108, 95% CI: [−0.281, −0.026]); POF and negative emotions together mediate the self-efficacy–kinesiophobia correlation (= − 0.069, 95% CI: [−0.190, −0.019]). The 95% CIs for all three indirect effects did not include zero, signifying significant mediating effects. The total indirect effect of POF and negative emotions was −0.335, accounting for 57.56% of the total effect. These findings suggested that both POF and negative emotions play mediating roles between self-efficacy and kinesiophobia, and they can also exert indirect effects individually through either POF or negative emotions.

**Table 4:** Chain-mediating effect of postoperative fatigue and negative emotions on self-efficacy and kinesiophobia.

	Pathways	Product of coefficients		Bootstrapping BC 95% CI	Percentage of total effect (%)
		β	Boot SE		
Effect					
Total effect		−0.582	0.055	−0.697, −0.475	100
Direct effect	X → Y	−0.247	0.067	−0.403, −0.062	42.43%
Indirect effect	Total indirect effect	−0.335	0.072	−0.526, −0.231	57.56%
	X → M1 → Y	−0.159	0.053	−0.248, −0.052	27.32%
	X → M2 → Y	−0.108	0.064	−0.281, −0.026	18.56%
	X → M1 → M2 → Y	−0.069	0.043	−0.190, −0.019	11.86%

CI, confidence interval; SE, standard error; VIF, Variance Inflation Factor; X, self-efficacy; M1, postoperative fatigue; M2, negative emotions; Y, kinesiophobia.

## 4 Discussion

This study investigated the potential correlation between self-efficacy and kinesiophobia in postoperative patients with PLC, explicitly exploring the chain-mediating effect of POF and negative emotions on self-efficacy and kinesiophobia. The findings demonstrated that the average kinesiophobia score among postoperative patients with PLC was 29.48 ± 11.88, with 58.81% of patients experiencing kinesiophobia. In addition, significant correlations were observed among self-efficacy, POF, negative emotions, and kinesiophobia. Briefly, this study confirms the chain-mediating effect of POF and negative emotions on self-efficacy and kinesiophobia, thereby validating all research hypotheses.

### 4.1 Current status of self-efficacy and kinesiophobia in postoperative patients with PLC

The self-efficacy score of postoperative patients with PLC was 24.76 ± 10.21, suggesting a moderate level of self-efficacy and corroborating Zhou et al. (2024), who reported a self-efficacy score of 25.24 ± 4.45 in patients with prostate cancer. The relatively low self-efficacy levels in patients with cancer are attributable to the dual impact of the disease on physical and mental health, resulting in diminished psychosocial adaptability (Zhu et al., 2024). Surgical trauma and disease-related stress further lessen self-efficacy, adversely influencing treatment adherence, rehabilitation, and prognosis. Patients with low self-efficacy often lack confidence in recovery, viewing cancer as insurmountable and surgery as futile, which hampers their compliance with postoperative activities.

In this study, the average kinesiophobia score was 29.48 ± 11.88, suggesting moderate- to high-level kinesiophobia, with 187 of 318 patients (58.81%) experiencing kinesiophobia. Notably, this result is lower than that of Chen et al. (2023), probably because of the inclusion of only laparoscopic surgery patients in this study, which involves less trauma. Both cancer and surgery profoundly affect patients’ physical and psychological states, manifesting as declined physical function, fatigue, pain, weakened immunity, and malnutrition, along with anxiety, depression, and fear. Hoedjes et al. (2022) demonstrated that over half of the studies reviewed reported a self-efficacy–lifestyle changes correlation in cancer survivors. Thus, healthcare providers should focus on enhancing self-efficacy and addressing kinesiophobia in postoperative patients with PLC to improve rehabilitation outcomes.

### 4.2 Direct impact of self-efficacy on kinesiophobia in postoperative patients with PLC

This study established a significant negative correlation between self-efficacy and kinesiophobia (*r* = −0.544, *p* < 0.001), corroborating Chen et al. (2025). Self-efficacy, as a psychological factor, plays a key role in disease recovery. Patients with higher self-efficacy display better stress management, disease control, and confidence in the recovery, yielding better-quality health behaviors (Zhang et al., 2021; Piao et al., 2022). Notably, self-efficacy correlates closely with adherence to self-exercise. Patients with high self-efficacy approach postoperative activities positively, define goals for mobilization, and persevere in functional exercises, thereby decreasing the probability of kinesiophobia (Tao et al., 2020; Li et al., 2024). Conversely, patients with low self-efficacy tend to embrace negative coping strategies when experiencing physical discomfort, aggravating their kinesiophobia (Chen et al., 2024). Hence, healthcare providers should focus on improving patients’ self-efficacy to decrease kinesiophobia and enhance rehabilitation outcomes.

### 4.3 Mediating role of POF between self-efficacy and kinesiophobia

This study established that POF mediates the self-efficacy and kinesiophobia correlation. Precisely, patients’ confidence in postoperative recovery directly affects their level of fatigue, in turn affecting their perception of kinesiophobia. Prior research suggests that when the ICFS-10 score exceeds 24, patients are considerably affected by POF, both physically and psychologically (Xu et al., 2021). Severe fatigue often causes physical weakness, emotional distress, and avoidance of physical activity, thereby exacerbating the risk of kinesiophobia (Cigdem Karacay et al., 2023). Self-efficacy indirectly affects cancer patients’ quality of life through fatigue (Adame et al., 2023). Patients with higher self-efficacy perceive less fatigue, demonstrate better adherence to rehabilitation exercises, and have lower levels of kinesiophobia. Despite advancements in enhanced recovery after surgery (ERAS) protocols, POF remains a pressing issue in patients with PLC. Therefore, healthcare providers should prioritize fatigue management through dietary guidance, regular routines, and psychological interventions, such as cognitive behavioral therapy and mindfulness-based stress reduction, to lessen fatigue and minimize kinesiophobia.

### 4.4 Mediating role of negative emotions between self-efficacy and kinesiophobia

This study demonstrated that negative emotions (e.g., anxiety and depression) markedly mediate the self-efficacy and kinesiophobia correlation. Patients with low self-efficacy tend to experience negative emotions, which worsen their kinesiophobia. Li et al. (2022) reported that self-efficacy indirectly affects kinesiophobia through mindfulness, which also correlates with emotional states. Patients with PLC often experience severe anxiety and depression because of high treatment costs, recurrence rates, mortality rates, and reduced quality of life (Sercu, 2022); these negative emotions diminish patients’ willingness to engage in early mobilization, further worsening kinesiophobia. Hence, healthcare providers should address patients’ emotional states through regular assessments, peer support groups, and success stories to improve self-efficacy and decrease kinesiophobia.

### 4.5 Chain-mediating effect of POF and negative emotions on self-efficacy and kinesiophobia

This study established that POF and negative emotions jointly mediate the self-efficacy and kinesiophobia correlation. The total indirect effect of these mediators accounted for 57.56% of the total effect, suggesting that declined self-efficacy aggravates fatigue and negative emotions, thereby increasing kinesiophobia. Fatigue and negative emotions often coexist and interact, affecting patients’ physical and psychological states (Nie, 2024; Xiao et al., 2024). Hara et al. (2022) reported that fatigue and psychological factors mediate the correlation between self-efficacy and physical function in cancer survivors. Moreover, interventions like multidisciplinary exercise programs (Fernandez-Rodriguez et al., 2023), pain neuroscience education (Martínez-Miranda et al., 2024), and cognitive functional therapy (Ahmad et al., 2023) have been established to decrease kinesiophobia and enhance patients’ emotional states, fatigue levels, and quality of life. Hence, healthcare providers should adopt a holistic approach, addressing both physical and emotional aspects of recovery to increase self-efficacy and decrease kinesiophobia.

### 4.6 Limitations

With a cross-sectional design, this study has several limitations worth acknowledging. First, while the online data collection method enhanced research efficiency, it may have led to the exclusion of digitally underserved populations. Moreover, recruiting participants exclusively from a single province could reduce the generalizability of the results to other regions or countries. Therefore, future studies should widen the sample size and include multiple regions. Second, the dynamic changes in kinesiophobia and its influencing factors were not explored. Thus, longitudinal studies are needed to examine the long-term effects of these factors on patients’ psychological states. Third, intervention strategies were not developed. Hence, future research should design and validate scientifically robust interventions to improve protocols for recovery in cancer care. Fourth, the outcome of early mobilization, a central concern, was not measured directly. It is suggested that future studies include direct measures of early mobilization. Fifth, the self-reported psychological assessments may have been subject to measurement bias due to subjective responses, interference by acute pain, and time-dependent postoperative variations in patient status. Future investigations should address these variables. Finally, this study did not address issues such as pain scores, type of anesthesia, psychiatric history, or social support, and more comprehensive investigations should be undertaken in the future.

## 5 Conclusion

This study establishes that kinesiophobia is prevalent among postoperative patients with PLC, with self-efficacy, POF, and negative emotions markedly influencing its severity. In addition, both POF and negative emotions exert a chain-mediating effect on self-efficacy and kinesiophobia. Briefly, this study offers a scientific basis for clinical interventions aimed at minimizing kinesiophobia, boosting patients’ confidence in recovery, and augmenting postoperative quality of life. By addressing POF and negative emotions, healthcare providers can help patients attain better physical and psychological outcomes.

## Data Availability

The original contributions presented in the study are included in the article/supplementary material, further inquiries can be directed to the corresponding authors.
